# Effectiveness of PMTCT Programme at Mogwase Health Centre, South Africa

**DOI:** 10.1186/1742-4690-9-S1-P114

**Published:** 2012-05-25

**Authors:** Iryna Chaparanganda

**Affiliations:** 1 University of KwaZulu Natal, South Africa

## Introduction

Today, HIV/AIDS-related conditions are a major contributor to childhood morbidity and mortality in South Africa. The PMTCT Unit, Antenatal Clinic (ANC) at Mogwase Health Centre (MHC), North West (NW) Province in South Africa runs a prevention mother-to-child transmission (PMTCT) programme aimed at reducing the number of HIV-infected babies born to HIV-positive mothers. The PMTCT Unit also undertakes polymerase chain reaction (PCR) test for surrounding clinics.

## Materials and methods

The abstract analyses the effects of PMTCT programme within the 2010 period. 626 (100%) pregnant women booked during this period were tested for HIV with pre- and post-testing counselling. 200 (31.9%) of them were tested positive. 117 (58.5%) of the HIV-positive women received dual therapy (AZT from 14 weeks of pregnancy (before 01.04.2010 – from 28 weeks) and sdNVP at onset of labour) and 83 (41.5%) were initiated on highly active antiretroviral therapy (HAART) (CD4 ≤350 cells/mm3 (before 01.04.2010 - ≤200 cells/mm3) or stage 4) – 200 (100%) in total. 200 (100%) of HIV-positive mothers also were counselled on safe infant feeding at least once.

## Results

133 HIV-exposed babies were born at the clinic, and 130 (97.7%) of them received nevirapine syrup whilst 3 (2.3%) did not receive and transferred to hospital. PCR tests around 6 weeks were done on 161 babies inclusive of those from other clinics. 4 (2.48%) infants got infected with HIV through MTCT of which, 1 (25%) was transferred from another clinic; 1 (25%) was from a late booked mother, and 2 (50%) from unbooked mothers.

## Conclusion

Although more effort is needed to encourage prevention of unplanned pregnancies, early pregnancy testing, and early booking at ANC site PMTCT programmes can be effectively implemented in rural clinics in South Africa, reducing the number of HIV-infected babies born to HIV-positive mothers.

## Source

ANC register. MHC, NW Province, South Africa.

PMTCT register. MHC, NW Province, South Africa.

Dispensing book. MHC, NW Province, South Africa.

PCR register. MHC, NW Province, South Africa.

